# Body Size Distribution of the Dinosaurs

**DOI:** 10.1371/journal.pone.0051925

**Published:** 2012-12-19

**Authors:** Eoin J. O’Gorman, David W. E. Hone

**Affiliations:** 1 School of Biological and Chemical Sciences, Queen Mary University of London, London, United Kingdom; 2 School of Biology and Environmental Science, Science Centre West, University College Dublin, Belfield, Dublin, Ireland; University of Pennsylvania, United States of America

## Abstract

The distribution of species body size is critically important for determining resource use within a group or clade. It is widely known that non-avian dinosaurs were the largest creatures to roam the Earth. There is, however, little understanding of how maximum species body size was distributed among the dinosaurs. Do they share a similar distribution to modern day vertebrate groups in spite of their large size, or did they exhibit fundamentally different distributions due to unique evolutionary pressures and adaptations? Here, we address this question by comparing the distribution of maximum species body size for dinosaurs to an extensive set of extant and extinct vertebrate groups. We also examine the body size distribution of dinosaurs by various sub-groups, time periods and formations. We find that dinosaurs exhibit a strong skew towards larger species, in direct contrast to modern day vertebrates. This pattern is not solely an artefact of bias in the fossil record, as demonstrated by contrasting distributions in two major extinct groups and supports the hypothesis that dinosaurs exhibited a fundamentally different life history strategy to other terrestrial vertebrates. A disparity in the size distribution of the herbivorous Ornithischia and Sauropodomorpha and the largely carnivorous Theropoda suggests that this pattern may have been a product of a divergence in evolutionary strategies: herbivorous dinosaurs rapidly evolved large size to escape predation by carnivores and maximise digestive efficiency; carnivores had sufficient resources among juvenile dinosaurs and non-dinosaurian prey to achieve optimal success at smaller body size.

## Introduction

The mass of an organism is fundamental to its biology, affecting physiology, ecology, metabolism and more [1], [2]. Knowledge of the mass of an adult individual, and by extension the species or genus to which it belongs, can therefore provide important information about the taxon in question. Much effort has thus been devoted to estimating the mass of the extinct non-avian dinosaurs (hereafter simply dinosaurs). As a group they are especially interesting as they feature numerous multi-ton taxa and include the largest terrestrial animals of all time [3]. Large size evolved early on in the Dinosauria, with multi-ton sauropodomorphs and basal sauropods appearing in the Late Triassic, and even the earliest dinosaurs show evidence for rapid growth [4]. However, while much research has been devoted to both mass estimates of dinosaurs (e.g. [5], [6]) and changes in body size (e.g. [7], [8], [9]), very limited attention has been paid to the distribution of dinosaur body size (but see [10], [11]), especially in the context of ecological implications [12], [13]. Dinosaurs may feature species that were considerably greater in maximum size to those of modern or other extinct animals, but this may only relate to the absolute size of a given taxon, rather than representing a fundamentally different distribution of body sizes within an entire group or clade.

The pattern of body size distribution is critically important for determining resource use: there is more usable space for small animals, so small-bodied species should be more prevalent in nature as they can better subdivide the habitat and co-exist in larger numbers [14], [15]. This phenomenon is highlighted by a skew towards small-sized species in many terrestrial groups [16], particularly mammals [17], [18], [19] and birds [20], [21], [22]. However, the positively-skewed distribution of these groups becomes less clear at the order level [18], [23] and at smaller spatial scales [19], [24]. This may be a product of small sample size [14], but it suggests that positively-skewed distributions are broad-ranging patterns, attributable to higher levels of taxonomic organisation at large biogeographical scales.

Maurer *et al*. [19] have also demonstrated that small body size is promoted by speciation, while extinctions are biased towards larger body size, leading to a higher probability of positively-skewed size distributions. These results were based on the models of McKinney [25], who suggested that, if most clades originate at small size, there is a lower limit on diversification toward small size, with size increases more likely. It has been shown that this lower constraint on species body size is a key factor driving the positively-skewed size distributions so often observed in nature [14]. The skew towards smaller species has also been linked to an optimum body size for a species based on the difference between assimilation and respiration [26], or energy that can be allocated to growth and reproduction. More recently, the concept of a size distribution around a common optimum for a taxon [26] has been rejected in favour of distributions of optimal sizes, different for each species and dependent on mortality and productivity [27], [28]. The latter phenomenon has been shown to produce a high prevalence of positively-skewed size distributions in simulated models, with occasional occurrence of negative skew [29], [30]. There is still, however, much uncertainty surrounding the mechanisms that lead to these exceptions to the rule.

As palaeontologists rely on modern analogues to inform our understanding of extinct ecologies, it is important to determine if dinosaurian size distributions were fundamentally similar or different to modern-day vertebrate groups. Here, we address this issue by comparing the body size distribution of dinosaurs to other known extant and extinct vertebrate groups. We also explore subdivisions of size distributions in dinosaurs by major clades, time periods and formations to tease apart the possible factors that facilitated the observed patterns.

## Methods

Extensive datasets of maximum species body size were collated from the literature for eight major animal groups: extant birds, reptiles, amphibians, fish and terrestrial mammals and extinct dinosaurs and pterosaurs and Cenozoic terrestrial mammals. These categories represent the major vertebrate groups, forming a logical point of comparison.

**Figure 1:**
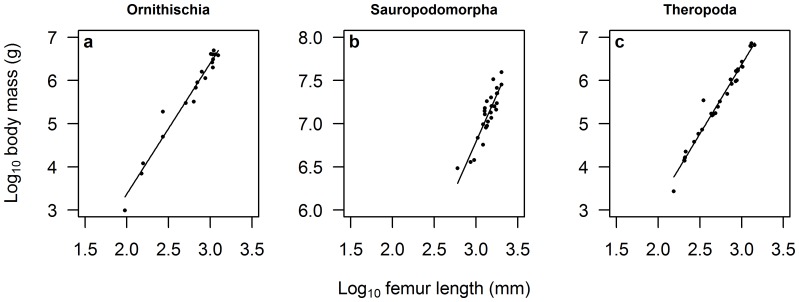
Log_10_(femur length)-log_10_(body mass) relationships for three major dinosaur clades: (a) Ornithischia (*y* = 3.0587*x*−2.7042; *r* ^2^ = 0.93), (b) Sauropodomorpha (*y* = 2.3459*x*−0.2935; *r* ^2^ = 0.73) and (c) Theropoda (*y* = 3.1854*x*−3.1840; *r* ^2^ = 0.97). The data sources for these relationships are shown in Table 1.

All dinosaur body masses were estimated from femur length-body mass relationships established during the study. Length-weight relationships were drawn separately for each of three clades (see Fig. 1): Ornithischia (19 data points, *r* ^2^ = 0.93), Sauropodomorpha (27 data points, *r* ^2^ = 0.73) and Theropoda (31 data points, *r* ^2^ = 0.97). The data collected to construct these relationships were the result of an extensive literature search spanning 41 separate publications and consist of all dependable published mass estimates for which a femur length could also be obtained (see Table 1). While some studies call into question the accuracy of volumetric models [31], these represent the best estimates of dinosaur body mass currently available. Femur lengths were acquired from the literature and museum specimens for a total of 329 out of approximately 1,350 dinosaur species (24% completeness). In cases where there were several individual femur length measurements available for a species, we chose to take the maximum femur length. While the use of limb bone circumference has been recommended for estimating mass [32], the combined data on femur lengths and body mass estimates were far more extensive. Additionally, a strong correlation (*r* ^2^ = 0.94) has been shown between femur length and diameter (a component of circumference) from a sample of 221 dinosaur individuals [33].

**Table 1:** Dinosaur taxa constituting the femur length-body mass relationships shown in Fig. 1, along with specimen numbers (where available), mass (in kg), femur length (FL in mm), source of mass measurement and reference to the paper containing the FL and mass estimate.

Clade	Genus	Species	Specimen	Mass (kg)	FL (mm)	Source	Reference
Ornithischia	*Anatosaurus* *	*copei* *	AMNH 5730	4000	1150	Limb bone scaling	[70]
Ornithischia	*Bactrosaurus*	*johnsoni*	AMNH 6553	1588.9	790.5	Polynomial	[6], [71]
Ornithischia	*Corythosaurus*	*casuarius*	AMNH 5240	3078.5	1080	Polynomial	[6], [72]
Ornithischia	*Edmontosaurus*	*annectens*	USNM 2414	3990.8	1068.5	Polynomial	[6], [72]
Ornithischia	*Edmontosaurus*	*regalis*	NMC 2289	3800	1245	Limb bone scaling	[70]
Ornithischia	*Gasparinisaura*	*cincosaltensis*	MUCPc-208	0.98	94.7	Polynomial	[6], [73]
Ornithischia	*Huayangosaurus*	*taibaii*	ZDM T7001	301.4	510	Polynomial	[6], [74]
Ornithischia	*Hypacrosaurus*	*altispinus*	NMC 8501	2000	1074	Limb bone scaling	[70]
Ornithischia	*Hypsilophodon*	*foxii*	NHM R196	7	150	Polynomial	[6], [75], [76]
Ornithischia	*Iguanodon*	*atherfieldensis*	NHM R5764	678.4	670	Polynomial	[6], [77]
Ornithischia	*Kentrosaurus*	*	HMN	321.1	633	3D Slicing	[6], [78]
Ornithischia	*Leptoceratops*	*gracilis*	NMC 8889	190	270	Limb bone scaling	[70]
Ornithischia	*Muttaburrasaurus*	*langdoni*	QM F6140	4100.4	1015	Polynomial	[6], [79]
Ornithischia	*Parkosaurus*	*warreni*	ROM 804	50	270	Limb bone scaling	[70]
Ornithischia	*Psittacosaurus*	*mongoliensis*	AMNH 6253	12.1	157	Polynomial	[6], [80]
Ornithischia	*Sauropelta*	*edwardsi*	AMNH 3036	902.9	700	Polynomial	[6], [81]
Ornithischia	*Stegosaurus*	*armatus*	USNM 4934	2610.6	1053	Polynomial	[6], [75], [76]
Ornithischia	*Triceratops*	*prorsus*	USNM 4842	4964	1104	Polynomial	[6], [82]
Ornithischia	*Tuojiangosaurus*	*multispinus*	CV00209	1134.3	875	Polynomial	[6], [83]
Sauropodomorpha	*Alamosaurus*	*sanjuanensis*		32663	1610	Growth lines	[84]
Sauropodomorpha	*Amargasaurus*	*cazaui*		6852.9	1050	Polynomial	[6], [85]
Sauropodomorpha	*Apatosaurus*	*		17273	1785	3D Slicing	[86]
Sauropodomorpha	*Apatosaurus*	*excelsus*		25952	1775	Growth lines	[84]
Sauropodomorpha	*Apatosaurus*	*louisae*	CM 3018	22407.2	1785	Polynomial	[6], [87]
Sauropodomorpha	*Barosaurus*	*		20039.5	1520	Polynomial	[6], [87]
Sauropodomorpha	*Brachiosaurus*	*altithorax*	FCM	28264.6	2030	Polynomial	[6], [88]
Sauropodomorpha	*Brachiosaurus* *	*brancai* *		39500	2028	Displacement	[89], [90]
Sauropodomorpha	*Camarasaurus*	*lewisi*	BYU 9047	11652.2	1525	Polynomial	[6], [87]
Sauropodomorpha	*Camarasaurus*	*supremus*		9300	1341	Displacement	[89], [90]
Sauropodomorpha	*Cetiosaurus*	*oxoniensis*		15900	1660	Displacement	[89], [90]
Sauropodomorpha	*Dicraeosaurus*	*hansemanni*		5700	1220	Displacement	[89], [90]
Sauropodomorpha	*Diplodocus*	*		13421	1506	3D Slicing	[5], [91]
Sauropodomorpha	*Diplodocus*	*carnegiei*		16000	1540	Displacement	[89], [90]
Sauropodomorpha	*Euhelopus*	*zdanskyi*		3800	955	Displacement	[90], [92]
Sauropodomorpha	*Haplocanthosaurus*	*		14528.6	1745	Polynomial	[6], [87]
Sauropodomorpha	*Haplocanthosaurus*	*priscus*		12800	1275	Displacement	[90], [92]
Sauropodomorpha	*Janenschia*	*robustus*		14029	1270	Growth lines	[84]
Sauropodomorpha	*Jobaria*	*tiguidensis*		22448	1800	3D Slicing	[93], [94]
Sauropodomorpha	*Mamenchisaurus*	*hochuanensis*		18169.7	1350	Polynomial	[6], [87], [95]
Sauropodomorpha	*Mamenchisaurus*	*hochuanensis*		15100	1275	Displacement	[89], [90]
Sauropodomorpha	Northampton*	sauropod*		9000	1320	Growth lines	[84]
Sauropodomorpha	*Omeisaurus*	*tianfuensis*		9800	1215	Displacement	[89], [90]
Sauropodomorpha	*Opisthocoelicaudia*	*skarzynskii*	ZPAL MgD-I/48	10522.2	1395	Polynomial	[6], [96]
Sauropodomorpha	*Patagosaurus*	*		9435.4	1360	Polynomial	[6], [87]
Sauropodomorpha	*Riojasaurus*	*		3038.7	600	Polynomial	[6], [97]
Sauropodomorpha	*Shunosaurus*	*lii*		3600	865	Displacement	[89], [90]
Theropoda	*Afrovenator*	*abakensis*	UCOBA1	826.6	760	Polynomial	[6], [98]
Theropoda	*Albertosaurus*	*		1685	905	Displacement	[99]
Theropoda	*Allosaurus*	*fragilis*		1620	874	Displacement	[99]
Theropoda	*Allosaurus*	*fragilis*	USMN 4734	952	850	Polynomial	[6], [100]
Theropoda	*Anserimimus*	*		170	433	Displacement	[99]
Theropoda	*Avimimus*	*portentosus*		14	205	Displacement	[89], [101], [102]
Theropoda	*Carnotaurus*	*sastrei*		2070	1030	Displacement	[89], [92], [103]
Theropoda	*Coelophysis*	*bauri*	AMNH FR 7223	16	209	Polynomial	[6], [102]
Theropoda	*Daspletosaurus*	*		2700	1006	Displacement	[99]
Theropoda	*Deinonychus*	*antirrhopus*	MCZ 4371	73	336	Displacement	[89], [102]
Theropoda	*Deltadromeus*	*agilis*	SGM-Din2	1048.9	740	Polynomial	[6], [104]
Theropoda	*Dilophosaurus*	*wetherilli*	UCMP 37302	325	551	Displacement	[99]
Theropoda	*Dromiceiomimus*	*		160	454	Displacement	[99]
Theropoda	*Elaphrosaurus*	*bambergi*	HMN dd	245	519	Displacement	[99]
Theropoda	*Eoraptor*	*lunensis*	PVSJ 512	2.7	154	Polynomial	[6], [105]
Theropoda	*Gallimimus*	*bullatus*	G.I.DPS 100/11	490	673	Displacement	[99]
Theropoda	*Gallimimus*	*bullatus*		38	270	Displacement	[89]
Theropoda	*Giganotosaurus*	*carolinii*	MUCPv-CH-1	6594.8	1430	Polynomial	[6], [106]
Theropoda	*Gorgosaurus*	*libratus*	TMP ?	1815	905	Displacement	[89]
Theropoda	*Herrerasaurus*	*ischigualastensis*		145	345	Displacement	[89], [102]
Theropoda	*Ornitholestes*	*hermanni*	AMNH 587	16.5	210	Displacement	[99]
Theropoda	*Ornithomimus*	*edmontonensis*	TMP ?	155	443	Displacement	[99]
Theropoda	*Oviraptor*	*philoceratops*		58	303	Displacement	[99]
Theropoda	*Saurornitholestes*	*langstoni*	TMP 88.121.39	22.5	214	Displacement	[99]
Theropoda	*Sinraptor*	*dongi*	TMP 90.300.1	1700	884	Displacement	[99]
Theropoda	*Sinraptor*	*dongi*	IVPP 10600	1009	876	Polynomial	[6], [107]
Theropoda	*Struthiomimus*	*altus*	AMNH 5339	175	486	Displacement	[99]
Theropoda	*Syntarsus*	*rhodesiensis*	QG/1	13.8	208	Polynomial	[6], [102]
Theropoda	*Tarbosaurus*	*		1650	854	Displacement	[99]
Theropoda	*Tyrannosaurus*	*rex*	CM 9780 (AMNH 5027)	6300	1273	Displacement	[99]
Theropoda	*Tyrannosaurus*	*rex*		7224	1314.5	3D Slicing	[5], [108]

Note that just 77 dinosaur species were identified in the literature with combined femur length and body mass estimates. Maximum femur lengths from a total of 329 species were used in the exploration of dinosaur body size distributions and these data are available on request from the authors.

* Note that *Anatosaurus copei* is now identified as *Edmontosaurus annectens* and *Brachiosaurus brancai* is now known as *Giraffatitan brancai*. In this table, we report the species names as listed in the original referenced publication for ease of cross referencing. This includes a number of dinosaur genera that do not contain species names in the original paper.

All bird data were extracted from Dunning’s 2008 handbook of avian body masses [34]. We chose to take the maximum body mass listed for each species, irrespective of the sex of the bird. These measurements constitute 9,381 out of approximately 10,000 bird species (94% completeness).

Reptile data were collated from a number of sources. Snout-vent lengths (SVL) for 4,874 lizard species were taken from Shai Meiri’s dataset [35]. Here, maximum SVL is seen as a good measure of the size potential in a population and is tightly correlated with mean adult SVL and SVL at sexual maturity [35], [36]. Lizard body masses were obtained using the SVL-mass allometries listed in Table 2 of Meiri’s 2010 publication [37]. Body mass data for a further 1,330 reptile species were obtained from Guyer and Boback’s online published dataset [38]. This included 1,030 snake species, 260 turtle species, 22 crocodilian species and a further 18 lizard species. Snakes were measured as maximum total length (TL) and converted to body mass using the TL-mass allometry listed in Pough’s 1980 publication [39]. Turtles were measured as maximum carapace length (CL) and converted to body mass using the CL-mass allometry listed in Pough’s 1980 publication [39]. Crocodiles were measured as maximum TL and converted to body mass using the TL-mass allometry listed in Table 3 of Farlow *et al*.’s 2005 publication [40]. Body masses for the two existing species of tuatara were taken from two recent publications [41], [42]. This resulted in body mass estimates for a total of 6,206 out of approximately 8,700 reptile species (71% completeness).

**Table 2:** Exploration of body size distributions for major vertebrate groups, dinosaur clades, time periods and formations.

Comments	Category	Skewness	Location of modes on *x*-axis of body size distribution					Lilliefors *D*	*p* value
Major vertebrate groups	Dinosaurs	−0.758	6.3					0.105	<0.001
(see Figure 2)	Birds	0.837	1.3	4.6	5.2			0.091	<0.001
	Reptiles	1.077	0.9	6.1				0.109	<0.001
	Amphibians	1.140	0.0	2.8	4.2			0.082	<0.001
	Fish	0.180	−1.6	1.5	1.9	2.4		0.021	<0.001
	Extant Mammals	0.906	1.5	4.8				0.118	<0.001
	Pterosaurs	0.226	3.0	4.5				0.117	0.084
	Extinct Mammals	0.333	2.0	4.7				0.089	<0.001
Major dinosaur clades	Ornithischia	−0.909	6.1					0.157	<0.001
(see Figure 4)	Sauropodomorpha	−1.501	7.1					0.161	<0.001
	Theropoda	−0.305	5.3					0.084	0.076
Major time periods	Late Triassic	−0.432	4.3	5.6	6.4			0.195	0.029
(see Figure 5)	Early Jurassic	0.071	4.1					0.143	0.354
	Middle Jurassic	−1.369	6.6					0.182	0.015
	Late Jurassic	−1.294	2.3	6.0	7.2			0.142	0.008
	Early Cretaceous	−0.089	3.8	6.0				0.114	0.052
	Late Cretaceous	−0.950	2.3	3.6	4.7	5.7	6.5	0.119	<0.001
Major Formations	Morrison	−0.558	7.2					0.223	0.003
(see Figure 6)	Dinosaur Park	−0.697	4.2	6.5				0.170	0.070

Values are given for skewness of the distribution, location of modes in the distribution, Lilliefors *D* statistic and the *p* value showing significant difference from a normal distribution.

Amphibian data were also obtained from Guyer and Boback’s online published dataset [38]. This included 1,424 anuran species, 244 Caudata and 101 Gymnophiona for a total of 1,769 out of approximately 6,500 species (37% completeness).

All fish data were collated from FishBase [43]. Fish body masses were calculated from maximum fish lengths (a mixture of total lengths, standard lengths and fork lengths) and their corresponding length-weight relationships. This resulted in body mass estimates for a total of 11,994 out of approximately 32,000 fish species (37% completeness).

Extant mammal body masses were taken from Smith *et al*.’s 2003 data paper [44], which provides body mass estimates for a total of 4,061 out of approximately 5,488 mammal species (74% completeness). Note that we considered only fully or predominantly terrestrial mammals. As such all chiropterans, cetaceans, sirenians and pinnipeds were excluded from this dataset.

Frequency distributions of maximum species body size were plotted from these data for each group, with size bins of 0.2 width on a log_10_ scale. A combination of kernel density estimation and smoothed bootstrap resampling (based on 1000 randomisations) was used to examine the modality of these body size distributions. This procedure (described in detail in [45], [46]) tests whether a distribution with *k+1* modes fits significantly better than a distribution with *k* modes, thus determining the optimum modality of the data. Other arbitrary techniques (e.g. [47]) typically overestimate the number of modes and gaps in body size distributions [45], [48]. The location of each mode was recorded relative to the *x*-axis. A measure of skewness was also calculated for each distribution as *g*_1_ = *m*_3_/*m*_2_^3/2^, where *m*_3_ is the sample third central moment and *m*_2_ is the sample variance (after [49]). To determine if the distribution was significantly skewed, it was tested against normality using Lilliefors (Kolmogorov-Smirnov) test. The body size distribution of dinosaurs was also compared to all other groups using the Kolmogorov-Smirnov test.

To investigate the influence of taphonomic bias in the fossil record, body size distributions were also explored for extinct pterosaurs and Cenozoic mammals. Here, the existence of a similar pattern in other extinct groups would be convincing evidence for fossil bias. Pterosaurs are the sister-taxon to the dinosauromorphs and, like the dinosaurs, originated in the Late Triassic and went extinct at the end of the Cretaceous, occupying numerous common ecosystems. Pterosaur body mass estimates were taken from wingspan data in Ross Elgin’s appendix for the forthcoming *Pterosauria* book [50]. This gave a total of 50 species. While this is a small dataset, it encompasses approximately one third of known pterosaur species. Mass estimates were calculated from a wingspan-weight formula in [51]. Cenozoic mammals provide a well-sampled clade of fossilised terrestrial taxa and form an obvious point of comparison to extant mammals. Cenozoic mammal body masses were taken from John Alroy’s online paleobiology database [52] used in Clauset and Erwin’s 2008 publication [53]. This gave a total of 2,034 species. As for the extant mammals, we considered only fully or predominantly terrestrial mammals. Pterosaur and Cenozoic mammal data were analysed as described above for the other vertebrate groups. The body size distribution of extant and Cenozoic mammals were also compared using the Kolmogorov-Smirnov test.

To evaluate the consistency of observed patterns in the body size distribution of dinosaurs, the data were reanalysed after sub-dividing by clades, time periods and formations. Three major clades were employed in this analysis: Ornithischia, Sauropodomorpha and Theropoda, with 143, 86 and 100 data points, respectively. Six time periods were used: Late Triassic, Early, Middle and Late Jurassic, and Early and Late Cretaceous which used 23, 21, 31, 58, 61 and 135 data points, respectively. Finally, two major rock formations also had a sufficient number of species to be utilised: the Late Jurassic Morrison Formation in the western United States of America and the Late Cretaceous Dinosaur Park Formation in Alberta, Canada, each of which used 24 data points. Similar metrics to those described above were obtained for these sub-divisions (but plotted with size bins of 0.5 width on a log_10_ scale where the number of data points was less than 100). All analyses were performed with R 2.14.0 (R Development Core Team 2011).

## Results

**Figure 2:**
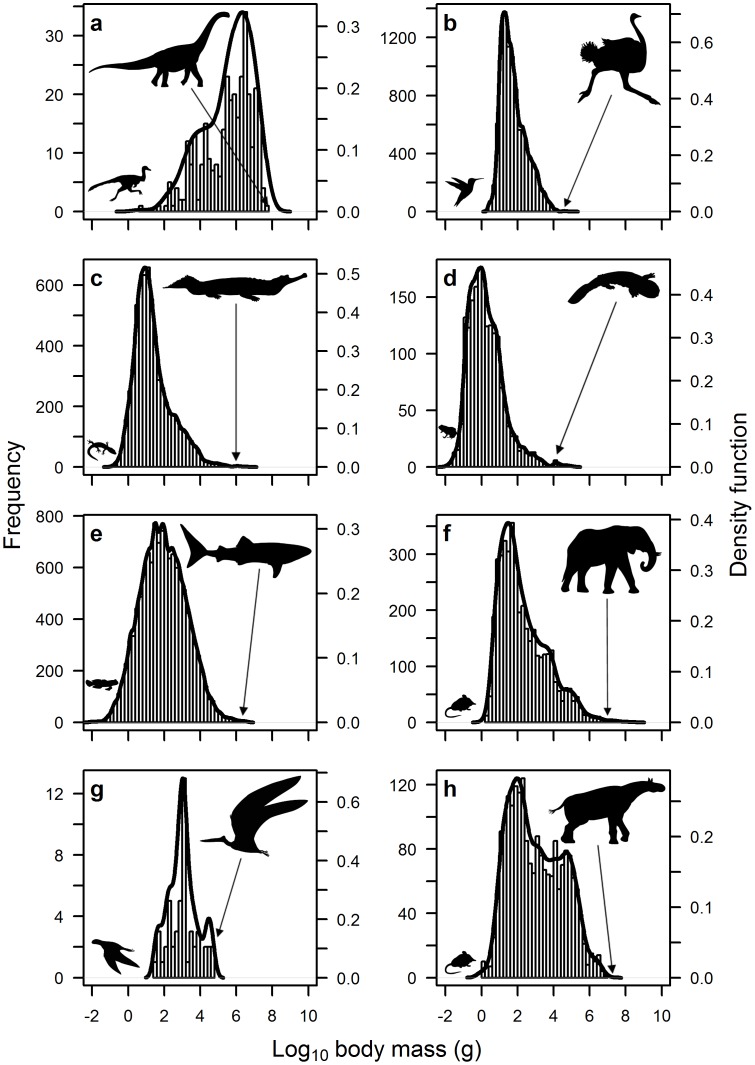
Frequency distribution of species body size for eight different animal groups: (*a*) extinct dinosaurs; (*b*) extant birds; (*c*) extant reptiles; (*d*) extant amphibians; (*e*) extant fish; (*f*) extant mammals; (*g*) extinct pterosaurs; and (*h*) Cenozoic mammals. Note that all distributions are positively-skewed except for dinosaurs, which are markedly negatively-skewed (see Table 2). A combination of kernel density estimation and smoothed bootstrap resampling was used to test for optimum modality of the body size distributions. Silhouettes of the largest and smallest animal in each group are also shown (provided by Matt van Rooijen).

Dinosaurs exhibit a unimodal negatively-skewed frequency distribution of maximum species body size, which is significantly different from a normal distribution (Lilliefors test: *D* = 0.105, *p*<0.001; see Fig. 2*a* and Table 2). This is in contrast to all other major extant groups, i.e. birds, reptiles, amphibians, fish and terrestrial mammals, which exhibit positively-skewed frequency distributions that are significantly different from a normal distribution (Lilliefors test: *p*<0.001; see Fig. 2*b–f* and Table 2). Reptiles and extant mammals are characterised by a bimodal positively-skewed distribution (Fig. 2*c, f* and Table 2), with the second peak in reptiles occurring at very large body size due to the large mass of the Crocodilia relative to all other groups. Birds and amphibians are distinctly positively-skewed, but with a distribution exhibiting several modes (Fig. 2*b,d* and Table 2). The distribution of maximum fish species body size more closely resembles a bell-shaped curve, but is still positively-skewed and significantly different from a normal distribution, with several modes (Fig. 2*e* and Table 2). The body size distribution of dinosaurs is also significantly different from all other groups (Kolmogorov-Smirnov test: *p*<0.001).

**Figure 3:**
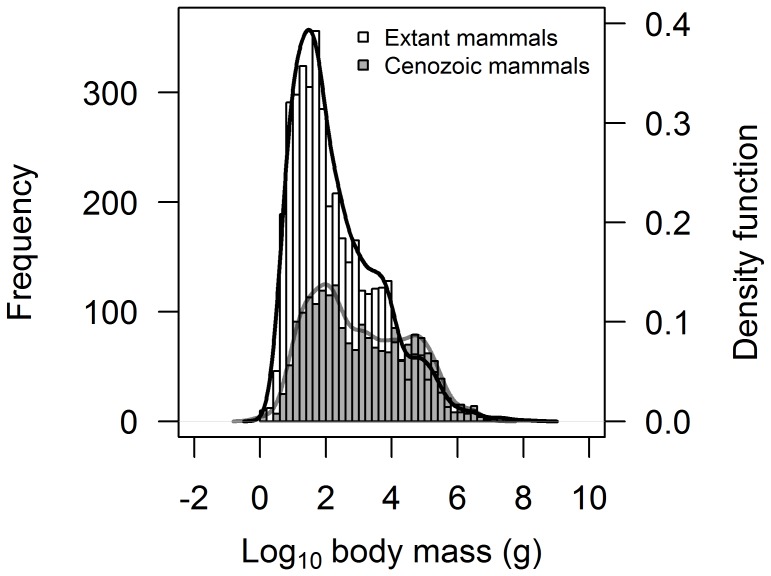
Frequency distribution of maximum species body size for Cenozoic mammals (in grey) overlaid on the distribution for extant mammals (in white). Curve fitting is based on a combination of kernel density estimation and smoothed bootstrap resampling. The figure clearly highlights the reduced frequency of small-bodied species in the Cenozoic mammal dataset, while the frequency of large-bodied species is comparable between both datasets.

The exploration of other fossilised taxa, the extinct pterosaurs and Cenozoic mammals, revealed that both these groups have positively-skewed distributions of maximum species body size, in contrast to the dinosaurs (Fig. 2*g–h* and Table 2). The body size distribution for pterosaurs is not significantly different from a normal distribution (Lilliefors test: *D* = 0.117, *p* = 0.084; see Table 2). However, the existing dataset for pterosaur species body mass is very limited (*n* = 50), so these trends should be interpreted with caution. The Cenozoic mammals are characterised by markedly fewer small species compared to extant mammals as evidenced by the truncated peak around a body mass of log_10_(2) g (see Fig. 2*h* and Fig. 3). Additionally, the body size distribution of these two groups are significantly different from each other (Kolmogorov-Smirnov test: *D* = 0.218, *p*<0.001). This provides evidence of taphonomic bias against the discovery of smaller species in the fossil record and yet the distribution of Cenozoic mammals is still distinctly positively-skewed and significantly different from a normal distribution (Lilliefors test: *D* = 0.089, *p*<0.001; see Table 2).

**Figure 4:**
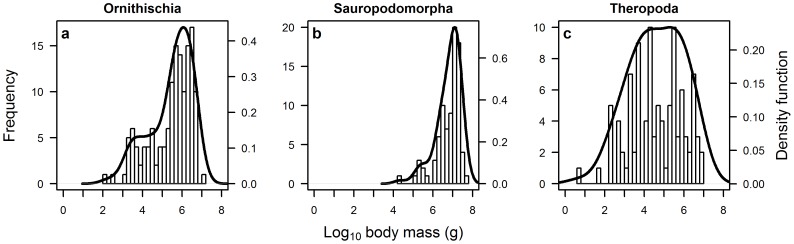
Frequency distribution of species body size for three major dinosaur clades: (*a*) Ornithischia; (*b*) Sauropodomorpha; and (*c*) Theropoda. The Sauropodomorpha and Ornithischia are significantly negatively-skewed, while the Theropoda exhibit a bell-shaped distribution (see Table 2). All three clades are best fitted by unimodal distributions.

The negatively-skewed distribution of maximum dinosaur species body mass was only found to be consistent for two of the three major clades. Here, both the Ornithischia and Sauropodomorpha exhibit markedly negatively-skewed unimodal distributions, which are significantly different from a normal distribution (Lilliefors test: *p*<0.001; see Fig. 4*a–b* and Table 2). While the body size distribution of Theropoda is somewhat negatively-skewed, it does not differ significantly from a normal distribution (Lilliefors test: *D* = 0.084, *p* = 0.076; see Fig. 4*c* and Table 2).

**Figure 5:**
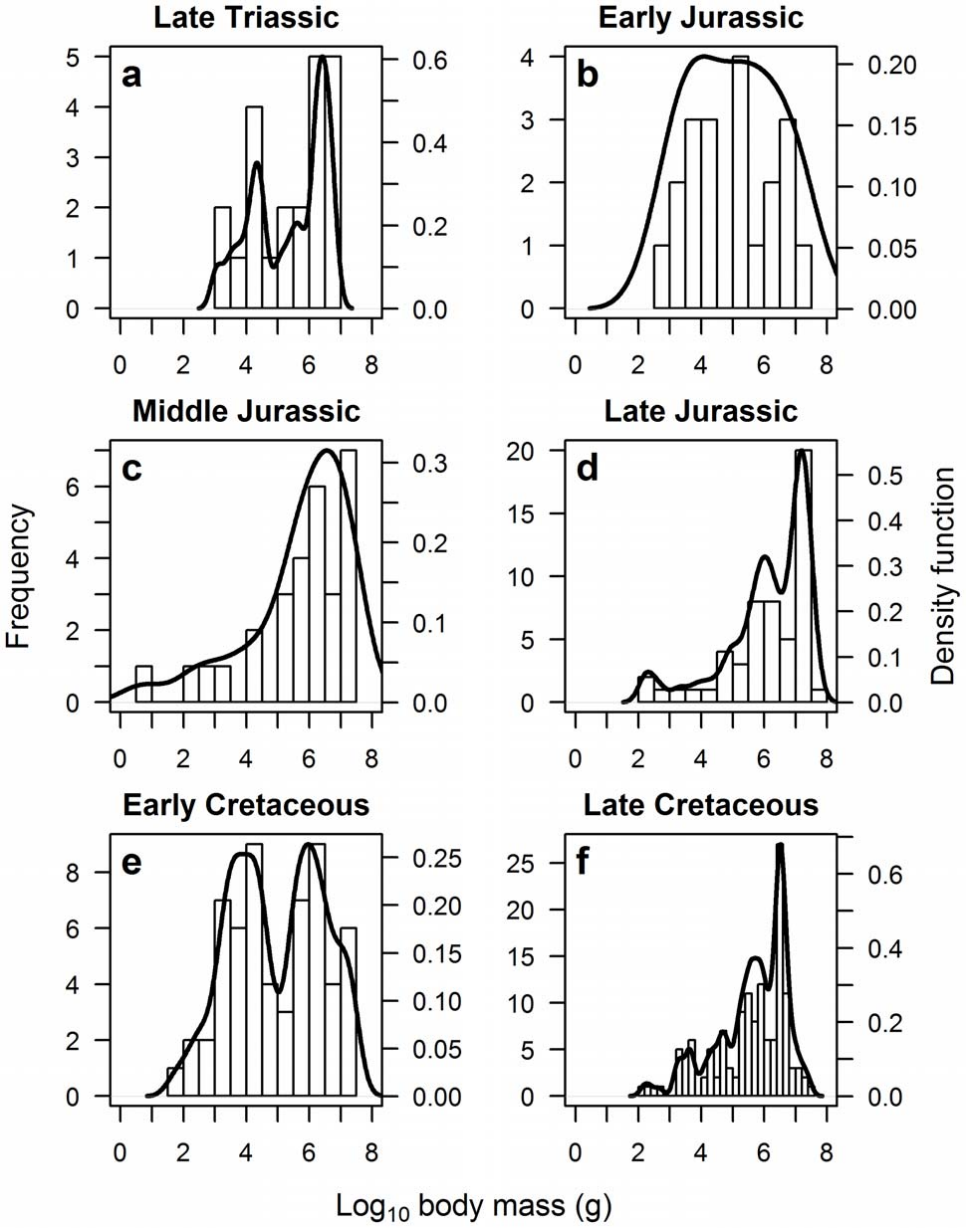
Frequency distribution of dinosaur species body size for six major time periods: (*a*) Late Triassic; (*b*) Early Jurassic; (*c*) Middle Jurassic; (*d*) Late Jurassic; (*e*) Early Cretaceous; and (*f*) Late Cretaceous. The Early and Middle Jurassic were best fitted by unimodal distributions; the Early Cretaceous by a bimodal distribution. The Late Triassic, Jurassic and Cretaceous were all best fitted by negatively-skewed multi-modal distributions (see Table 2).

The distribution of maximum dinosaur species body size was only found to be distinctly negatively-skewed towards the end of each major time period. Here, the Late Triassic, Late Jurassic and Late Cretaceous periods all display multi-modal negatively-skewed distributions, which are significantly different from a normal distribution (Lilliefors test: *p*<0.029; see Fig. 5*a,d,f* and Table 2). The additional modes may be partly explained by the reduced number of data points constituting these analyses. Dinosaur body size was also skewed towards larger species in the Middle Jurassic, with a unimodal distribution that is significantly different from a normal distribution (Lilliefors test: *D* = 0.182, *p* = 0.015; see Fig. 5*c* and Table 2). The Early Jurassic and Early Cretaceous periods showed many smaller as well as larger species of dinosaur, with unimodal and bimodal distributions, respectively, which are not significantly different from a normal distribution (Lilliefors test: *p*>0.052; see Fig. 5*b,e* and Table 2).

**Figure 6:**
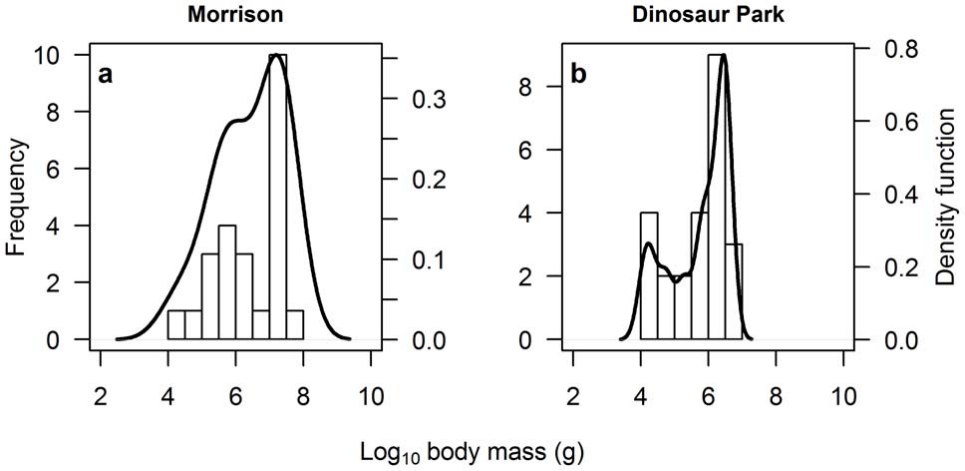
Frequency distribution of dinosaur species body size for two major formations: (*a*) the Morrison and (*b*) Dinosaur Park. Both formations showed negatively-skewed distributions, with the Morrison formation approximately unimodal and the Dinosaur Park formation best fitted by a bimodal distribution (see Table 2). These patterns should be interpreted with caution, however, due to the small number of data points for each formation.

Finally, the two formations of dinosaur fossils with sufficient data for sampling, the Morrison and Dinosaur Park, again demonstrated negatively-skewed distributions of maximum species body size, with unimodal and bimodal distributions respectively (see Fig. 6 and Table 2). The body size distribution of the Morrison was found to be significantly different from a normal distribution (Lilliefors test: *D* = 0.223, *p* = 0.003), while the distribution for Dinosaur Park exhibited no significant difference from normality (Lilliefors test: *D* = 0.170, *p* = 0.070). Again, we caution about the small number of data points making up these analyses.

## Discussion

Dinosaurs appear to be unique among vertebrates by demonstrating a strong skew in size distribution towards larger species. All other major extant vertebrate groups are dominated by a prevalence of smaller-bodied species (Fig. 2). Thus, it is not only absolute size, but also the size distribution that is skewed towards larger forms. While the fossil record suffers from a number of biases [10], [54], the distribution of dinosaurs here does not appear to be solely an artefact of the fossil record, as demonstrated by the similarity in positively-skewed data for extant and extinct mammals (see Fig. 3 and Table 2). Taphonomic processes are clearly at play, however, with a significant difference observed in the body size distribution of these two groups. Here, the peak in the distribution for large species remains largely unchanged in both data sets, while the peak for smaller species is suppressed and shifted to the right in Cenozoic mammals. Combined with existing knowledge of taphonomic biases in the dinosaur fossil record from a recent detailed study on the Dinosaur Park formation [10], this highlights the need to interpret the observed body size distribution for dinosaurs in Fig. 2*a* with caution.

However, it is also clear that taphonomic bias is unlikely to completely alter the interpretation of skewness of body size distributions. Brown *et al*. [10] identified a mass of 60 kg as marking the point below which taxa were vulnerable to being missed from the fossil record. In order to convert our overall dinosaur distribution dataset to match that of the extinct mammals, we would have to be missing around 90% of the non-avian dinosaurian diversity and all of it small (i.e. under 60 kg). To get our dinosaur distribution to match that of the extant mammals or birds, we would have to be missing 99.99% of diversity. Thus, we consider it implausible that taphonomic bias can be the sole force driving these results. Moreover, the pterosaurs (sister-taxon to the dinosauromorphs, living alongside them in the same environment and subject to similar conditions and taphonomic biases) display a more ‘typical’ vertebrate distribution in their body size (see Fig. 2*g*), suggesting the dinosaurian signal is genuinely unique.

If the evolution of large body size was a product of long exposure to a stable environment, we would expect a steady progression from skew towards small species, to a bell-shaped distribution of species body size, before finally developing the skew towards large body size we observe in Fig. 2*a*. It is interesting that smaller species were more prevalent in the Early Jurassic and Early Cretaceous periods (see Fig. 5*b, e*), in conjunction with large periods of species turnover in other groups [55]. By the end of both periods, dinosaurs exhibit a skew towards larger species once more (Fig. 5*d, f*), providing some evidence for periods of stability leading to the evolution of larger size. However, the presence of marked left skew in the Late Triassic, just after the emergence of dinosaurs as a novel clade (Fig. 5*a*), shows that this characteristic size distribution was acquired early in dinosaurian evolution and immediately became a fixture of dinosaur-dominated ecosystems, and indeed large sauropodomorphs are known from the Late Triassic [12], [56], [57]. There is also little evidence for a geographical bias in the prevalence of large body size in dinosaurs. Data from two species-rich formations, the Morrison and Dinosaur Park, both reveal a skew towards larger-bodied species (Fig. 6), although the data are too patchy to make a definitive judgement on this pattern. It should also be noted that other ecosystems may show different patterns (e.g. while untested here due to insufficient data, the Yixian Formation in China would appear to be dominated by smaller taxa).

Given the discrepancy between dinosaurs and all other vertebrates, the origin of this unusual body size distribution presumably lies in some major aspect of dinosaurian biology that distinguishes them from other taxa. It has been hypothesised that dinosaurs had a life history strategy unique to dominant terrestrial vertebrate clades [4]. Here, thanks to the small size at hatching and large size at adult, large dinosaurs grew through multiple orders of magnitude to reach adulthood. In consequence, they would have occupied multiple ecological niches during ontogeny and thus the apparent absence of small dinosaur species may in part be explained by the occupation of these niches by the juveniles of large species [4]. Furthermore, the size distribution of the theropods differs markedly from the ornithischians and sauropodomorphs (see Fig. 4). Most theropods were carnivorous (especially the larger forms e.g. tyrannosaurids, abelisaurids, allosauroids) and so their size could be considered contingent on the prey species available from the ranks of the herbivores. Notably, there were numerous small theropod species (Fig. 4*c*) and although theropods as a whole might be expected to preferentially target juvenile dinosaurs for their prey [58], non-dinosaurian prey (e.g. lizards, mammals) would also have been available for smaller theropods. Thus, there is a disparity in the mechanisms driving size strategies in the various clades. The largely carnivorous theropods had sufficient animal resources to achieve optimal success (sensu [14]) at lower body size. In contrast, the herbivorous sauropodomorphs and ornithischians may have achieved optimal success through rapidly growing to a large body size that was outside the optimal foraging range of likely theropod predators [59], [60], [61], and provided a more beneficial feeding strategy (see below).

Comments on the giant size of dinosaurs have understandably tended to focus on the sauropodomorphs. For example, Sander *et al*. [12] noted a different body size distribution for sauropodomorphs versus a dataset of theropods and ornithischians combined. However, when separated out as shown in Fig. 4, the ornithischians have a more sauropodomorph-like distribution. In attempting to explain this apparent discrepancy, Sander *et al*. [12] focused on unique features of sauropodomorph paleobiology that might have facilitated or driven such large sizes and size distribution for the clade, but we suggest that the features driving large body size in the dinosaurs are not exclusive to the Sauropodomorpha. The ornithischians also featured numerous large taxa (37% of known species with available body mass estimates were greater than one ton), many of which exceeded the smaller sauropodomorphs in size. Burness *et al*. [62] showed that the body masses of the largest sauropods and theropods exceeded that predicted by the area of the land they occupied, yet no ornithischians were analysed as part of this work and at least some of these would similarly exceed the expected values. Thus, while Sander *et al*. [12] make a convincing case for the uniqueness of the sauropods with respect to their great size, a number of their supposedly unique features were also present in the ornithischians and may have similarly affected this clade. Both include species that grew through five orders of magnitude, from a few kilos to over ten tons. Sauropodomorphs and ornithischians also had similar reproductive strategies, with both capable of laying 20 or more eggs in a single nest [63] and achieving rapid growth to large body size [64]. Thus, although there were factors that may have helped promote extreme large size in sauropods not seen in ornithischians, such as their avian-like respiratory system and light skeletons [12], the potential strategies for optimal success were likely similar overall in the ornithischians.

One of the most notable factors affecting the herbivorous sauropodomorphs and ornithischians is digestive efficiency. Gut volume increases linearly [65] and basal metabolism is a fractional power [66] of body weight. These relationships produce a metabolic requirement to gut capacity ratio that decreases with body size, thus increasing the proportion of digested food particles in larger herbivores [67]. It is thought that this relationship may have played a major role in overcoming energetic issues through the optimisation of nutritive value from energy-rich, but slow fermenting pre-angiosperm plants [68]. This could be as true for multi-ton ornithischians as sauropodomorphs, where large size would also lead to increased gut volume and by extension greater digestive time. The largely carnivorous theropods would not have benefited from the same gut retention strategy and thus may not have exhibited the same evolutionary necessity for extreme large size. But this begs the question, why have other major groups not evolved similar divergent strategies?

It may be too energetically costly for endotherms to maintain a very large body mass and there is a danger of overheating [69]. As such, it is more beneficial for birds and mammals to possess a relatively small body size. If larger dinosaurs were ectothermic or gigantothermic as has been proposed [12], they would not be constrained in this way. Reptiles, amphibians and fish are also ectothermic or gigantothermic, however, and thus may be expected to show a similar response to dinosaurs. The vast majority of modern day reptiles and amphibians are carnivorous and will not benefit from increased digestive efficiency at large size, as argued for the Theropoda. While many fish are also carnivores, there is a sizeable proportion of planktivores and herbivores. It is interesting then that the body size distribution of fish is not as distinctly skewed towards small species as for the other major extant groups (see Fig. 2*e* and Table 2). This may reveal a possible trend towards increased body size in response to digestive efficiency. Future studies should examine the body size distribution of herbivorous relative to carnivorous fish species to explore this possibility in more detail.

Thus, while the exact evolutionary pressures and anatomical exaptations that led to large body size in dinosaurs is still a matter for debate [12], [13], the data presented here suggest a body size distribution that is unique among known vertebrate groups (see Fig. 2). While taphonomic processes may play a role in accentuating the negative skew of this distribution, there is also evidence for a divergence in optimal size strategies for the carnivorous theropod clade and the herbivorous sauropodomorph and ornithischian clades. This divergence is most likely related to the availability of small resources for the predatory theropods and the need to escape predation and maximise digestive efficiency in the herbivorous clades.
