# Correction: Body Size Distribution of the Dinosaurs

**DOI:** 10.1371/annotation/032ce6c0-22e3-4ae8-997a-40859bcb5609

**Published:** 2013-04-09

**Authors:** Eoin J. O’Gorman, David W. E. Hone

The equal contributions designation was erroneously omitted from the published manuscript. Authors Eoin J. O’Gorman and David W. E. Hone contributed equally to this work. 

